# Visual input to the mouse lateral posterior and posterior thalamic nuclei: photoreceptive origins and retinotopic order

**DOI:** 10.1113/JP271707

**Published:** 2016-03-31

**Authors:** Annette E. Allen, Christopher A. Procyk, Michael Howarth, Lauren Walmsley, Timothy M. Brown

**Affiliations:** ^1^ Faculty of Life SciencesUniversity of ManchesterUK

## Abstract

**Key points:**

- The lateral posterior and posterior thalamic nuclei have been implicated in aspects of visually guided behaviour and reflex responses to light, including those dependent on melanopsin photoreception.
- Here we investigated the extent and basic properties of visually evoked activity across the mouse lateral posterior and posterior thalamus.
- We show that a subset of retinal projections to these regions derive from melanopsin‐expressing retinal ganglion cells and find many cells that exhibit melanopsin‐dependent changes in firing.
- We also show that subsets of cells across these regions integrate signals from both eyes in various ways and that, within the lateral posterior thalamus, visual responses are retinotopically ordered.

**Abstract:**

In addition to the primary thalamocortical visual relay in the lateral geniculate nuclei, a number of other thalamic regions contribute to aspects of visual processing. Thus, the lateral posterior thalamic nuclei (LP/pulvinar) appear important for various functions including determining visual saliency, visually guided behaviours and, alongside dorsal portions of the posterior thalamic nuclei (Po), multisensory processing of information related to aversive stimuli. However, despite the growing importance of mice as a model for understanding visual system organisation, at present we know very little about the basic visual response properties of cells in the mouse LP or Po. Prompted by earlier suggestions that melanopsin photoreception might be important for certain functions of these nuclei, we first employ specific viral tracing to show that a subset of retinal projections to the LP derive from melanopsin‐expressing retinal ganglion cells. We next use multielectrode electrophysiology to demonstrate that LP and dorsal Po cells exhibit a variety of responses to simple visual stimuli including two distinct classes that express melanopsin‐dependent changes in firing (together comprising ∼25% of neurons we recorded). We also show that subgroups of LP/Po cells integrate signals from both eyes in various ways and that, within the LP, visual responses are retinotopically ordered. Together our data reveal a diverse population of visually responsive neurons across the LP and dorsal Po whose properties align with some of the established functions of these nuclei and suggest new possible routes through which melanopsin photoreception could contribute to reflex light responses and/or higher order visual processing.

## Key points

- The lateral posterior and posterior thalamic nuclei have been implicated in aspects of visually guided behaviour and reflex responses to light, including those dependent on melanopsin photoreception.
- Here we investigated the extent and basic properties of visually evoked activity across the mouse lateral posterior and posterior thalamus.
- We show that a subset of retinal projections to these regions derive from melanopsin‐expressing retinal ganglion cells and find many cells that exhibit melanopsin‐dependent changes in firing.
- We also show that subsets of cells across these regions integrate signals from both eyes in various ways and that, within the lateral posterior thalamus, visual responses are retinotopically ordered.

**Abbreviations**
APT: anterior pretectum
dLGN: lateral geniculate nuclei
LGN: lateral geniculate nuclei
LP: lateral posterior thalamic nuclei
mRGC: melanopsin‐expressing retinal ganglion cell
Po: posterior thalamic nuclei
RF: receptive field
SC: superior colliculus
V1: primary visual cortex
YFP: yellow fluorescent protein

## Introduction

In the mammalian visual system, the lateral geniculate nuclei (LGN) are the major thalamic relay providing input to primary visual cortex (V1), from which we build our conscious perception of the world (Nassi & Callaway, 2009). However, while lesions of V1 abolish visual perception, some ability to discriminate visual stimuli/perform visually guided behaviours persists in most mammals (Dean, 1981; Lomber, 2002; Prusky & Douglas, 2004; Leopold, 2012). This residual visual function is believed to involve the lateral posterior thalamic nuclei (LP; also known as the pulvinar), which integrates signals from the superior colliculus (SC), cortex and retina and communicates with both higher visual cortical areas and subcortical sites such as the amygdala (Kaas & Lyon, 2007; Day‐Brown *et al*. 2010; Tamietto *et al*. 2012). Accordingly, in the intact animal, the LP is believed to play important roles in determining the saliency of visual stimuli, processing motion‐related information and driving reflex responses to threatening/fear‐related stimuli (Morris *et al*. 1999; Grieve *et al*. 2000; Goossens *et al*. 2007; Wurtz *et al*. 2011; Van Le *et al*. 2013; Wei *et al*. 2015).

While it is relatively well established then that the LP plays several important roles within the visual system, our understanding of the functional properties of LP neurons is still relatively limited. This is particularly the case in mice, which have become an especially important model for studying visual system organisation and function (Huberman & Niell, 2011). Just as in other mammals, the mouse LP receives visual input from the retina (Morin & Studholme, 2014) and the SC (Gale & Murphy, 2014) and is reciprocally connected with several cortical areas (Tohmi *et al*. 2014). These cortical connections appear important for processing motion‐related information, as destruction of the SC results in impaired tuning properties in higher visual cortical areas (Tohmi *et al*. 2014), while projections to the amygdala drive defensive responses (freezing) to stimuli simulating airborne predators (Wei *et al*. 2015).

The LP is also implicated in multisensory processing (Baldwin *et al*. 2011) and one study has demonstrated that a subset of cells in the rat LP and underlying posterior thalamus (Po, another thalamic nucleus implicated in multisensory processing; Sewards & Sewards, 2002) integrate visual and nociceptive inputs (Noseda *et al*. 2010). Based on neuroanatomical tracing, this earlier study suggested that a portion of the retinal projections to the LP and dorsal Po may derive from melanopsin‐expressing intrinsically photosensitive retinal ganglion cells (mRGCs). This retinal projection to the LP/Po has been proposed as the route through which light exacerbates migraine pain. Although the presence of mRGC inputs to the LP/Po awaits definitive confirmation, in principle such mRGC inputs could also play broader roles, for example in modulating attention, light avoidance and/or freezing behaviour (Mrosovsky & Hattar, 2003; Johnson *et al*. 2010; Delwig *et al*. 2012). Indeed, light avoidance behaviour in neonatal mice is known to be associated with Fos induction in the Po (Delwig *et al*. 2012).

Accordingly, here we initially set out to confirm the extent to which melanopsin photoreception constitutes an important source of input to cells in the LP and dorsal Po. To this end, we first employed mRGC‐selective viral tracing in mice expressing Cre‐recombinase under control of the melanopsin promoter (Opn4^+/cre^; Ecker *et al*. 2010). We next employed electrophysiological recordings in mice with long‐wavelength shifted cone sensitivity (Opn1mw^R^; Smallwood *et al*. 2003), allowing us to isolate melanopsin‐dependent responses (Brown *et al*. 2010, 2012) and confirm their functional extent across the LP/Po. As the visual response properties of mouse LP/Po cells [sensitivity, occularity and receptive field (RF) size/position] are currently unknown, we finally aimed to determine whether such properties resembled those reported in other species and/or differed from those observed in other subcortical visual nuclei in the mouse.

## Methods

### Animals

All animal use was in accordance with the Animals, Scientific Procedures, Act of 1986 (UK) and received institutional ethics committee and UK Home Office approval. Wildtype (WT: C57/Bl6 background), Opn1mw^R^ (Smallwood *et al*. 2003) and Opn4^+/cre^ (Ecker *et al*. 2010) mice were housed under a 12 h dark/light cycle environment at a temperature of 22°C with food and water available *ad libitum*.

### Retinal projection tracing

Five *Opn4^+/cre^* mice were anaesthetised with an intraperitoneal injection of ketamine (100 mg kg^−1^) and xylazine (10 mg kg^−1^). Once anaesthetised, a drop of 1% tropicamide and 2.5% phenylephrine was applied to the left eye in order to dilate the pupil. A fine Hamilton needle (35 G; 8 mm) fitted to a 5 μl Hamilton glass syringe was then passed through the equator of the sclera and into the vitreous cavity whilst being careful to avoid the lens. The injection consisted of 3 μl of a floxed Brainbow virus (AAV9‐hEF1a‐lox.TagBFP‐lox.eYFP‐lox.WPRE‐hGH‐InvBYF; 10^13^ genomic particles ml^–1^; Vector Core, USA) with a 0.5 μl mixture of Heparinase III (250 units ml^−1^; Sigma Aldrich, Poole, UK) and Hyaluronan lyase (250 units ml^−1^; Sigma Aldrich). The injection was conducted slowly over the course of 1 min. Once complete, the needle was gently removed and a topical analgesic (0.25% bupivicaine) was applied to the injected eye.

After a 4 week incubation period, mice were transcardially perfused with 0.1 m PBS followed by 4% paraformaldehyde. Brains and retina were removed and post‐fixed in 4% paraformaldehyde before being cryoprotected in a 30% sucrose solution. Brains were sectioned at 120 μm on a freezing sledge microtome before undergoing immunohistochemistry for the enhanced yellow fluorescent protein (eYFP) protein.

In brief, sections were initially blocked in 10% donkey serum (Sigma Aldrich) for 3 h before being placed in primary antibody (chicken anti‐GFP; 1:1000; Abcam, Cambridge, MA, USA) for 3 days at 4°C. Sections were then subsequently washed before being incubated in a secondary antibody of Alexa‐488 conjugated donkey anti‐chicken (1:200; Jackson Immunoresearch, West Grove, PA, USA) for 12 h at 4°C. Sections were then placed onto glass slides and mounted with ProGold Diamond (Invitrogen, Paisley, UK). Images were collected on a Leica DM2500 microscope [with a CoolLED pE300 LED light source filtered through a Chroma L5 ET(k)/Chroma Y3 ET(k) filter set] using a Leica DFC365 FX camera.

Transduction efficiency for viral injections was determined by comparing the density eYFP‐labelled cells in the corresponding retinal whole mounts (*n* = 5) relative to the density of mRGCs reported previously (∼2000 per retina; Ecker *et al*. 2010). Extrapolating cell counts made across one quadrant of each injected retina, we estimated a mean (±SEM) transduction efficiency of 31 ± 6% (range: 16.4–46%). Based on the morphology/dendritic stratification of eYFP‐expressing cells, it appeared that this approach labelled both M1 and non‐M1 type mRGCs with no overt preference towards a particular cell class.

### *In vivo* neurophysiology

Urethane (1.55 g kg^−1^) anaesthetised adult male mice (50–100 days old) were prepared for stereotaxic surgery and insertion of multielectrode arrays as described previously (Howarth *et al*. 2014). Recording probes (A4×8‐5mm‐50‐200‐177; Neuronexus, Ann Arbor, MI, USA) consisting of four shanks (spaced 200 μm), each with eight recording sites (spaced 50 μm apart) were coated with fluorescent dye (CM‐DiI; Invitrogen) and inserted into the LP/Po region (2.3 mm caudal and 1.4 mm medial to bregma) at depths between 2.9 and 2.5 mm relative to the brain surface. In some experiments, after recording responses from the Po we then raised our electrode by 400 μm to record a second set of data from the overlying LP. No attempt was made to adjust probe position to detect any particular kind of visual response. Accordingly, we consider the distribution of cell types encountered in this study to be an essentially unbiased assessment.

After allowing 30 min for neural activity to stabilise following probe insertion, wideband neural signals were acquired using a Recorder64 system (Plexon, Dallas, TX, USA), amplified (×2000) and digitized at 40 kHz. Action potentials were discriminated from these signals offline as ‘virtual’‐tetrode waveforms as described previously (Howarth *et al*. 2014) using custom MATLAB scripts (Mathworks, Natick, MA, USA). In brief, data were high pass filtered in forward and reverse directions (300 Hz, 4th order Butterworth) and grouped as overlapping sets of linear tetrodes (three tetrodes covering the eight recording sites on each shank). Tetrode waveforms (40 samples per channel) were then discriminated by threshold crossing (typically 45–55μV) and sorted manually using commercial principal components‐based software (Offline sorter, Plexon). Single unit isolation was confirmed by reference to MANOVA F statistics, J3 and Davies‐Bouldin validity metrics (Offline sorter) and the presence of a distinct refractory period (>1.5 ms) in the interspike interval distribution. Particular care was taken to ensure that no cell was discriminated more than once on overlapping tetrodes (confirmed via cross‐correlogram analysis of unit firing).

### Visual stimuli and analysis

All light measurements were performed using a calibrated spectroradiometer (Bentham Instruments, Reading, UK).

#### Full field stimuli

For experiments in *Opn1mw^R^* mice, visual stimuli were delivered in a light‐proofed chamber using a custom built source (Cairn Research, Faversham, UK) consisting of independently controlled UV, blue and red LEDs (λ_max_: 365, 460, and 630 and 655 nm, respectively). Light was combined by a series of dichroic mirrors and focused onto a 5 mm diameter piece of opal diffusing glass (Edmund Optics, York, UK) positioned < 1 mm from the eye contralateral to the recording probe. LED intensity was controlled by a PC running LabView 8.6 (National Instruments, Austin, TX, USA).

In our initial experiments we applied 30 s of bright blue (460 nm) or red (655 nm) light steps from a background of darkness (providing 14.9 and 15.4 log photons cm^–2^ s^–1^, respectively, at the level of the cornea). By reference to the Govardovski nomograms (Govardovskii *et al*. 2000) and published values for mouse lens transmission (Jacobs & Williams, 2007) we chose these values to provide identical stimulation of *Opn1mw^R^* cones (λ_max_ = 556 nm, effective irradiance = 14.4 log photons cm^–2^ s^–1^). Under these conditions the effective photon flux of both stimuli is saturating for mouse rods [14.8 and 12.1 photons cm^–2^ s^–1^, respectively (Nathan *et al*. 2006; Brown *et al*. 2010, 2011 *a*)] and is largely subthreshold for evoking S‐cone opsin‐mediated responses (11.6 and 4.8 photons cm^–2^ s^–1^, respectively; see Allen *et al*. 2011), whereas the blue but not red stimulus is sufficiently bright to robustly activate melanopsin [14.9 and 11.2 photons cm^–2^ s^–1^, respectively (Berson *et al*. 2002; Wong *et al*. 2007; Brown *et al*. 2010, 2013)].

In subsequent experiments, we investigated the specific contributions of cones and melanopsin to visually evoked activity in the LP/Po by measuring responses to transitions between carefully calibrated pairs of polychromatic stimuli (Brown *et al*. 2012; Allen *et al*. 2014; Walmsley, 2015). The first stimulus pair selectively differed in apparent brightness for S‐ and *Opn1mw^R^* cone opsin (‘cone‐isolating’ − 78% Michelson) with minimal change in rod and melanopsin excitation (contrast = 7 and 1%, respectively). The second stimulus pair constituted a large shift in melanopsin excitation (‘melanopsin‐isolating’ − 92% Michelson) with negligible S‐ and *Opn1mw^R^* cone opsin contrast (<0.05%). Although this latter stimulus also constitutes a large change in rod excitation (85% Michelson), the high background light levels employed here (effective irradiance = 14 log photons cm^–2^ s^–1^) mean that rod intrusion is unlikely to contribute significantly to any responses (Nathan *et al*. 2006; Brown *et al*. 2012; Walmsley, 2015). Stimuli were presented as 5 s steps from low to high contrast, with transitions occurring smoothly over 40 ms.

For experiments in WT mice, full field visual stimuli were generated via two LEDs (λ_max_ 405 nm; half‐width: ± 7 nm; Thorlabs, Newton, NJ, USA) independently controlled via LabVIEW (National Instruments) and neutral density filter wheels (Thorlabs). Light was supplied to the subject via 7 mm diameter flexible fibre optic light guides (Edmund Optics), positioned 5 mm from each eye and enclosed within internally reflective plastic cones that fit snugly over each eye – preventing any off‐target effects due to scattered light.

To determine the relative magnitude and sensitivity of eye‐specific responses in LP/Po neurons, mice were maintained in darkness and 5 s light steps were applied in an interleaved fashion to contra‐ and/or ipsilateral eyes for a total of ten repeats at logarithmically increasing intensities spanning 9.8–15.8 log photons cm^–2^ s^–1^ (interstimulus interval 20–50 s depending on intensity). Our choice of 410 nm LED stimuli for these experiments was based on the fact that all mouse photoreceptors display similar sensitivity in this part of the spectrum. After correction for pre‐receptoral filtering (as above), effective photon fluxes for each mouse opsin were between 0.5 (M‐ and S‐cone opsins) and 0.3 log units (melanopsin) dimmer than the values reported above. Owing to these properties, our measures of sensitivity are not biased towards responses originating from any specific photoreceptor(s). Intensities reported here reflect effective irradiance for rod opsin, which is intermediate between cones and melanopsin (9.4–15.4 log photons cm^–2^ s^–1^).

Single cells were considered responsive when mean stimulus‐evoked firing rate exceeded the 95% confidence limits of pre‐stimulus activity. For measurements of response latency we calculated peristimulus histograms for the above stimuli (1 ms bin size, Gaussian smoothing: σ = 5 ms) and found the first bin that exceeded the 95% confidence limits of the prestimulus (0–1 s) firing activity. Values reported in the text represent the fastest response observed (usually evoked at the highest intensity tested). In the case of transient cells (which often exhibited changes in firing at both light on and light off), latency was defined as the fastest of the two components.

#### Spatially structured stimuli

For RF mapping, stimuli were delivered via an LCD display (width: 26.8 cm, height: 47.4 cm; Hanns‐G HE225DPB) angled at 45 deg from the vertical and placed at a distance of ∼21 cm (occupying ∼63 × 96 deg of visual angle), either directly in front of the animal or rotated by 90 deg and positioned laterally at an angle of 30 deg relative to the animal's midline. In most experiments we applied visual stimuli at both of these locations sequentially.

Stimuli were generated and controlled via MATLAB using the Psychophysics toolbox (Brainard, 1997; Pelli, 1997) and comprised white or black flashing bars (430 and 3.3 sc. cd m^–2^, respectively) superimposed on a background of the opposite polarity. The screen was divided into a 45 × 80 grid and vertical or horizontal bars (occupying five adjacent grid squares; ∼7 deg at horizontal and vertical meridians) appeared at each possible location (in random sequence) for 250 ms followed by a blank screen for 250 ms. Thus, there was considerable spatial overlap between possible bar positions. These stimuli were run for a total of eight repeats sequentially for each bar orientation, polarity and screen location. RF parameters were then determined separately for azimuth and elevation under all conditions by plotting the mean response to stimuli of appropriate polarity (100 ms epochs starting 35–125 ms after bar appearance) as a function of bar position. These were then fit with one‐dimensional Gaussians to estimate RF centre position and diameter (full width at half maximum). Values for diameter reported in the text represent the averages of estimates obtained using vertical and horizontal bars of the appropriate polarity. For calculations of visual angle, tangent correction was applied in the azimuthal direction only.

In some experiments we also employed a second set of ‘sparse noise’ stimuli as described previously (Piscopo *et al*. 2013; Howarth *et al*. 2014). These comprised white and black spots presented on a grey background at a density such that on average 16% of the screen area was covered at any one time. Spots were 2, 4, 8, 16 and 32 deg in diameter and presented at a density inversely proportional to their size such that, on average, each spot size occupied an equal fraction of the visual display. Under each viewing condition, 10 min sequences of these stimuli were applied at 4 frames s^–1^. Data were analysed by reverse correlation separately for black and white spots (to avoid averaging out ON/OFF responses of non‐linear cells); for each pixel of the screen we calculated the mean firing rate over the 250 ms frame duration (50 ms epochs) as a function of pixel intensity and subtracted the mean firing rates when that pixel was not covered by a spot. Where tested (*n* = 9 cells), RFs mapped using bars and sparse noise were in close agreement.

### Histology

At the end of each experiment, mice were perfused transcardially, the brain was removed and sectioned (100 μm) as described above and then mounted directly onto slides using Vectasheild (Vector Laboratories, Peterbourogh, UK). DiI‐labelled probe placements were then visualised under a fluorescence microscope (Olympus BX51) with appropriate filter sets. Resulting images were then scaled to account for shrinkage (based on the known distance between electrode shanks) and aligned with appropriate stereotaxic atlas figures (Paxinos, 2001) using the optic tract, LGN and hippocampus as landmarks. Anatomical locations of recorded cells were then estimated from these images, based on the projected location of the recording site where we observed the largest spike amplitudes. From our previous work (Howarth *et al*. 2014), we think it very unlikely that any isolated unit was located more than 50 μm away from the recording site where we observe the largest spike amplitudes. Cells detected at recording electrodes projected to lie within the LGN were excluded from all the analyses reported in the present study.

All the cells recorded in this study were located at coronal levels corresponding to mid‐caudal portions of the LP or underlying Po (corresponding to atlas sections between −1.94 and −2.54 mm relative to bregma). To increase sampling coverage for cell density/retinotopic analysis here we have mapped cell locations onto a single anatomical template of the LP (−2.3 mm relative to bregma), by adjusting medial–lateral coordinates to align the lateral borders of the LP at different rostrocaudal levels. To produce maps of cell type densities, we then calculated the relative proportion of each cell type using a moving circular window (radius 120 μm; step size 40 μm, bins containing fewer than ten cells were discarded) and smoothed the resulting maps by cubic spline interpolation. An essentially identical procedure was used to produce maps of LP retinotopic organisation, with the moving window used to calculate mean RF position in azimuth or elevation planes (here bins containing at least three cells were included in the analysis).

## Results

### mRGC projections to the LP

**Figure 1:**
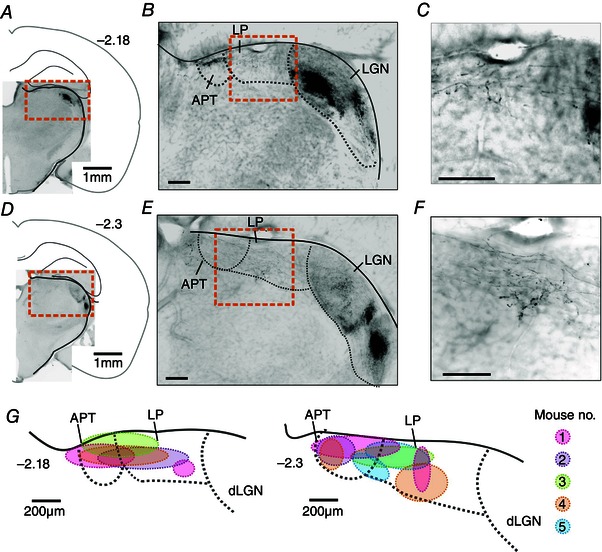
**Melanopsin ganglion cell projections to the lateral posterior thalamus** Anterograde tracing of melanopsin ganglion cells was achieved by injecting a virus containing floxed eYFP and BFP sequences (AAV‐ hEF1a.lox.TagBFP.lox.eYFP.lox.WPRE.hGH‐InvBYF) into the vitreous of *Opn4^cre/+^* mice. *A–C*, representative example of eYFP expression in LP and LGN at ∼−2.18 mm from bregma. *D–F*, representative example of eYFP expression in LP and LGN at ∼−2.30 mm from bregma. eYFP signal shown as black. *A* and *D*, whole thalamus; *B* and *E*, magnifications of orange box in *A* and *D*; *C* and *F*, magnifications of orange box in *B* and *E*. Scale bars = 200 μm. *G*, regions of mRGC terminal labelling identified in five *Opn4^cre/+^* mice receiving unilateral intravitreal viral injections.

Given the previous suggestion of a direct mRGC projection to the rat LP/Po (Noseda *et al*. 2010), our preliminary aim was to confirm the existence of this pathway in the mouse. To selectively label mRGC axons here we injected a virus containing floxed fluorescent protein sequences (AAV‐hEF1a.lox.TagBFP.lox.eYFP.lox.WPRE.hGH‐InvBYF) into the vitreous of *Opn4^+/cre^* mice. In transfected mRGCs, this virus should drive expression of yellow and blue fluorescent protein, enabling the central projection patterns of these ganglion cells to be identified. Accordingly, fluorescently labelled axons were visible in known targets of mRGCs (including the LGN; Fig. 1). In addition, labelled axons/terminals were visible in the contralateral LP, with a survey of five injected animals revealing that mRGC fibres/terminals could be found across broad regions of the central and medial aspects of the LP. In individual animals, mRGC innervation was often clustered to discrete regions, however (Fig. 1 *C*, *F*, *G*), presumably reflecting the incomplete viral transduction of mRGCs following intravitreal injection (estimated ∼30% efficiency).

### Basic visual response properties

**Figure 2:**
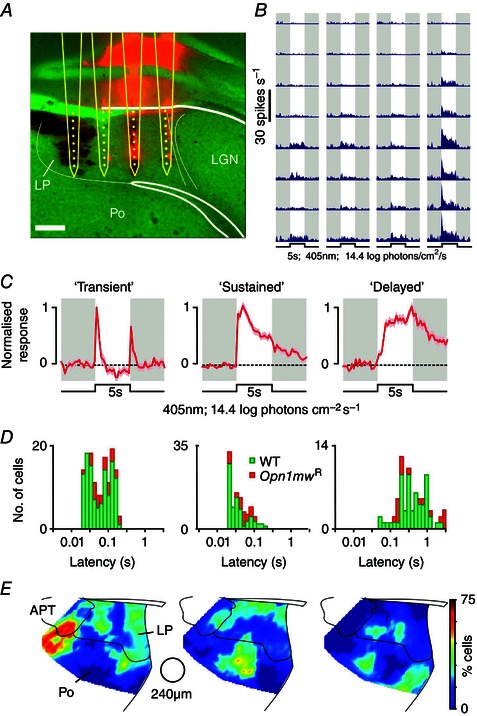
**Fast and slow visual responses across the lateral posterior and posterior thalamus** *A*, histological reconstruction of a multi‐electrode probe placement within the lateral posterior thalamus (LP). Probe shanks are marked with DiI (red fluorescence), light microscopic image is pseudocoloured green. Scale bar = 200 μm, Po = posterior thalamus, LGN = lateral geniculate nuclei. *B*, multi‐unit firing responses (at probe placement shown in *A*) evoked by a 5 s, 405 nm, light step producing an effective corneal irradiance of 14.4 log photons cm^–2^ s^–1^ at the contralateral eye. *C*, normalised mean (±SEM) change in firing for three classes of wild‐type (WT) LP/Po neuronal response to full field light steps as in *B* (*n* = 115, 74 and 58 for transient, sustained and delayed cells, respectively). *D*, response latency distributions for LP/Po cells from WT and *Opn1mw^R^* animals (*n* = 26, 29 and 16 for *Opn1mw^R^* transient, sustained and delayed cells). *E*, anatomical distribution of transient, sustained and delayed cells, showing the % of cells of each class binned using a moving circular window (240 μm diameter). Analysis based on a total of 526 WT and 162 *Opn1mw^R^* neurons (including 279 and 91 cells, respectively, that did not show overt responses to full field light steps). APT = anterior pretectum.

Having established that at least a portion of the retinal projections targeting the LP derive from mRGCs, we next sought to determine the anatomical extent of light‐evoked activity across this nucleus and the degree to which the visual response properties of individual cells were influenced by mRGC inputs. To this end, we extensively surveyed visually evoked activity across the LP and surrounding regions via extracellular recordings employing multisite (32 channel) silicon probes (Fig. 2 *A*, *B*). In total we performed 56 recordings (from 51 mice), from which we isolated 318 visually responsive cells (out of 688 in total). As explained below, to facilitate identification of melanopsin‐dependent responses, a subset of these recordings (*n* = 11) were performed in a mouse model (*Opn1mw^R^*) with long‐wavelength shifted M‐cone spectral sensitivity (Smallwood *et al*. 2003). The basic visual response properties of cells (*n* = 71) encountered in these *Opn1mw^R^* recordings were essentially identical to those of WT cells in every aspect that we tested, and accordingly we have combined the two datasets for further analysis where appropriate.

**Figure 3:**
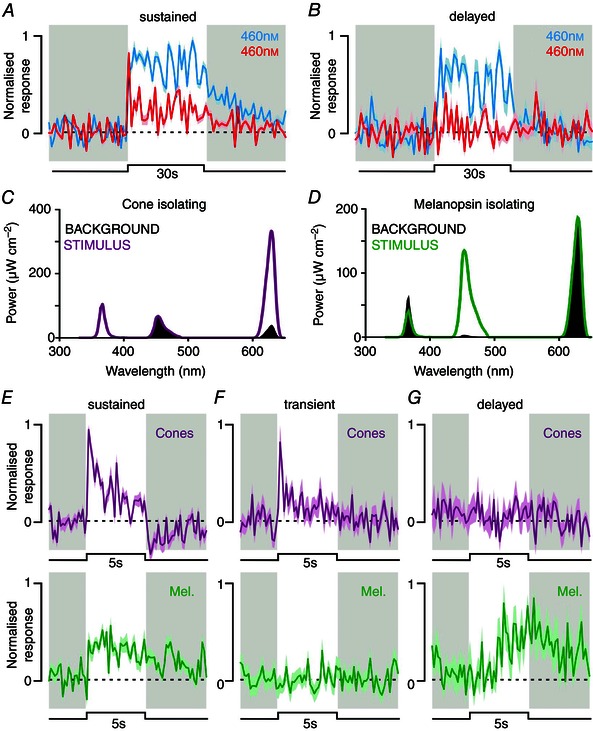
**Melanopsin contributions to lateral posterior and posterior thalamic visual responses** *A* and *B*, normalised mean (±SEM) change in firing for classes of LP/Po neuronal response to full field 460 nm (blue line; 30 s 14.9 log melanopsin‐effective photons cm^–2^ s^–1^, 14.4 log L‐cone‐effective photons cm^–2^ s^–1^) or 655 nm (red line; 30 s 11.2 log melanopsin‐effective photons cm^–2^ s^–1^, 14.4 log L‐cone‐effective photons cm^–2^ s^–1^) steps in *Opn1mw^R^* mice (*n* = 15 and 10 for sustained and delayed cells, respectively). *C* and *D*, spectral composition of cone opsin and melanopsin isolating stimuli; traces show background spectra, and purple/green lines show stimulus spectra (respectively). Steps from background to stimulus spectra result in 78% Michelson contrast for L‐ and S‐ cone opsins (*C*) or 93% Michelson contrast for Melanopsin (*D*). *E–G*, normalised mean (±SEM) change in firing for three classes of LP/Po neuronal response to 5 s full field cone‐ (upper panel) or melanopsin‐ (lower panel) isolating steps in *Opn1mw^R^* mice (*n* = 14, 17 and 6 for sustained, transient and delayed cells, respectively).

We started by assessing the responses of WT cells to bright full field light steps designed to robustly activate all known photoreceptor classes (5 s, 405 nm LED, 15.4 log photons cm^–2^ s^–1^). Based on the temporal profile of the resulting light response, visually responsive cells (*n* = 247/526 cells) could be clearly segregated into three classes (Fig. 2 *C*, *D*), analogous to those we have identified previously in other brain regions (Brown *et al*. 2010, 2011 *b*; Sakhi *et al*. 2014). The vast majority of visually responsive cells we encountered (*n* = 189/247) exhibited rapid changes in firing in response to light steps, whose latency (Fig. 2 *D*, media*n* = 45 ms, range = 21–220 ms) was broadly similar to that of visually responsive cells encountered in other primary retinorecipient nuclei such as the LGN (median = 40 ms, range = 23–190 ms; Howarth *et al*. 2014). Among these cells, more than half (*n* = 115/189) showed rapid increases in firing at light onset and/or offset (usually both) that decayed to baseline levels within 1–2 s following a change in light levels (Fig. 2 *C*, ‘transient), while the remaining cells with fast responses (*n* = 74/189) maintained elevated firing rates throughout the duration of the light step and for several seconds following return to darkness (Fig. 2 *C*, ‘sustained’). The third class of cells we identified (Fig. 2 *C*, ‘delayed’) also exhibited prolonged elevations in firing rate in response to full field light steps but here the onset of the light response was typically extremely sluggish (Fig. 2 *D*; median = 385 ms, range = 52–3863 ms) and firing increased steadily throughout the duration of light exposure. Subsequent experiments in *Opn1mw^R^* animals revealed a similar mix of cellular responses (*n* = 26, 29 and 16 for transient, sustained and delayed cells, respectively) following bright blue light steps (Fig. 2 *D* and Fig. 3, 5–30 s duration, 460 nm, 14.8 log photons cm^–2^ s^–1^).

Interestingly, we observed a quite marked anatomical segregation of the various classes of visually sensitive neurons described above (Fig. 2 *E*). Transient cells were especially prominent along the lateral and medial margins of the LP and ventral tip of the anterior pretectum (APT), whereas sustained cells were most commonly found in two areas: one along the dorsal margin of LP and the other in a more ventral area mainly situated in the Po with a small incursion in the ventral part of LP. By contrast, delayed cells appeared to surround the ventral pool of sustained cells, most commonly being found in the ventral LP or Po (Fig. 1).

### Photoreceptor contributions

A well‐established feature of melanopsin‐dependent visual responses at the level of the retina or brain is a sustained and persistent response to bright light exposure (Berson *et al*. 2002; Brown *et al*. 2010; Allen *et al*. 2011). Based on the properties of LP neurons we observed here we hypothesised that melanopsin photoreception could make a substantial contribution to the responses of sustained and delayed, but not transient, cells. To test this hypothesis we next employed, in subsets of recordings from *Opn1mw^R^* mice, two independent approaches previously shown to be effective at isolating response components dependent on melanopsin phototransduction (Brown *et al*. 2010, 2011 *b*, 2012; Allen *et al*. 2011, 2014; Walmsley, 2015).

We first investigated responses to full field blue (460 nm) and red (655 nm) light steps matched to produce identical activation of the red‐shifted cone opsin in *Opn1mw^R^* mice (effective irradiance = 14.4 log photons cm^–2^ s^–1^). As these stimuli differ substantially in their ability to activate melanopsin phototransduction (but are both sufficiently bright to maximally activate rods; see Methods), we would expect any cell receiving melanopsin input to show greatly enhanced responses to the blue stimulus. Consistent with this reasoning, both sustained (Fig. 3 *A*) and delayed cells (Fig. 3 *B*) exhibited significantly greater firing in response to 460 *vs*. 655 nm stimuli (mean ± SEM; sustained cells: 11.8 ± 2.4 *vs*. 4.3 ± 1.3, *n* = 15; delayed cells: 4.1 ± 1.6 *vs*. 0.5 ± 0.3, *n* = 10; paired *t*‐test, both *P* < 0.05). The population tested here included cells from both the LP (6 sustained, 4 delayed) and Po (9 sustained, 6 delayed) and statistical analysis on a single cell basis indicated that all but one of these cells (Po, sustained) fired significantly more spikes to 460 *vs*. 655 nm. Moreover, in line with our previous work in other brain regions (Brown *et al*. 2011 *b*), we found that whereas sustained cells still exhibited robustly detectable responses to the long‐wavelength stimuli (consistent with a substantial rod/cone input), delayed cells did not. None of the cells identified in this subset of recordings (*n* = 2 mice) was classified as transient.

While the data above are certainly consistent with the possibility that melanopsin phototransduction makes a major contribution to the responses of sustained and delayed cells, there is one important caveat to this conclusion: visual inputs from RGCs whose responses were strongly biased in favour of UV‐sensitive cone opsin inputs could also result in larger responses to short‐ *vs*. long‐wavelength stimuli (e.g. see Allen *et al*. 2011). Accordingly, in subsequent experiments we used an alternative approach which allowed us to selectively manipulate melanopsin and cone inputs to LP cells (Brown *et al*. 2012; Allen *et al*. 2014; Walmsley, 2015). To this end, working under rod saturating conditions, we next investigated responses to transitions between carefully calibrated pairs of polychromatic stimuli (see Methods) designed to either: (1) differ substantially in effective excitation of UV‐sensitive and *Opn1mw^R^* cone opsins (Fig. 3 *C*, 78% Michelson contrast) with negligible contrast for melanopsin or (2) produce a large change in excitation of melanopsin (Fig. 3 *D*, 93% Michelson contrast) with negligible contrast for the two cone opsins.

Consistent with our data above, we found that the group of sustained cells tested (Fig. 3 *E*; *n* = 14, 7 in LP, 7 in Po) showed significant increases in firing rate in response to both melanopsin‐ and cone‐selective increases in brightness (group means ± SEM: 2.8 ± 0.9 and 3.0 ± 0.6, respectively; paired *t*‐test, both *P* < 0.01; all individual cells exhibited significant increases in firing to cone stimuli and all but one to melanopsin stimuli). Moreover, in line with our initial hypothesis that transient cells were unlikely to receive melanopsin input, we found that this group of cells (Fig. 3 *F*, *n* = 17) responded to cone‐isolating stimuli but lacked detectable responses to changes in melanopsin activation (1.1 ± 0.4 and 0.1 ± 0.1, *P* < 0.05 and *P* = 0.92, respectively). Conversely, delayed cells (Fig. 3 *G*) exhibited significant increases in firing (2 LP cells and 3/4 Po cells at *P* < 0.05) in response to melanopsin‐selective brightness increments and all lacked detectable responses to selective activation of cones (group means ± SEM: 2.4 ± 1.1 and −0.2 ± 0.3, *P* < 0.05 and *P* = 0.5, respectively).

In summary, our data indicate that the distinct temporal profiles of the various classes of neurons we detect throughout the LP and surrounding regions are at least partially explained by differing photoreceptor inputs.

### Binocular integration and sensitivity

Regions of the LP are known to receive input from both the contralateral and the ipsilateral retina (Morin & Studholme, 2014) and are also known to receive synaptic input from other visual centres that could convey signals from either retina (Gale & Murphy, 2014; Tohmi *et al*. 2014). Accordingly, we next set out to determine whether any of the various classes of visually sensitive neurons we encountered above exhibited binocular responses and/or were differentially sensitive to eye‐specific inputs.

**Figure 4:**
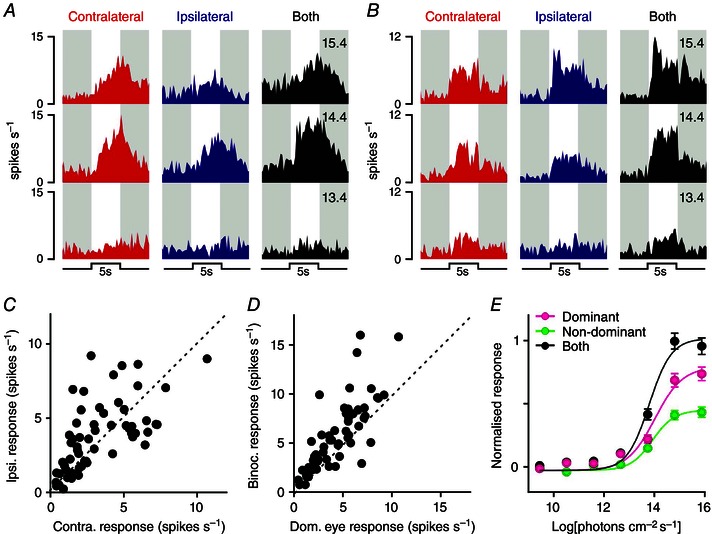
**Delayed cells integrate visual signals from both eyes** *A* and *B*, example responses of two delayed cells following full field light steps applied to the contralateral, ipsilateral or both eyes (5 s, 405 nm LED, 13.4–15.4 log photons cm^–2^ s^–1^). *C*, distribution of contralateral *vs*. ipsilateral eye‐evoked response amplitude (change in mean spike rate during 5 s light step relative to 5 s preceeding) for delayed cells in WT mice (*n* = 58). All cells exhibited responses to both eyes, with variable preference towards contra‐ or ipsilateral eye. *D*, distribution of both *vs*. dominant eye‐only‐evoked responses (measured as in *C*). Binocular responses were reliably larger than those evoked by the dominant eye alone. *E*, normalised mean (±SEM) change in firing for delayed cells in WT mice evoked by 9.4–15.4 log photons cm^–2^ s^–1^ light steps applied to the dominant, non‐dominant or both eyes. Note that across the population, sensitivity to light is extremely low and that binocular facilitation is associated with an increase in maximal response with no change in sensitivity (four‐parameter sigmoid curve fits with F‐tests for difference in EC_50_/Hill slope or maxima, *P* = 0.397 and < 0.0001, respectively).

We first examined the properties of delayed cells, using diffuse light steps applied to the ipsilateral and/or contralateral eye across a range of intensities (5 s, 405 nm, 9.4–15.4 log photons cm^–2^ s^–1^). In common with the properties of an analogous group of cells we previously observed in the lateral habenula (Sakhi *et al*. 2014), delayed cells encountered across the LP/Po region (*n* = 58 WT cells including 27 in the LP and 31 in Po) reliably exhibited relatively low visual sensitivity (minimum response threshold = 12.4 log photons cm^–2^ s^–1^) and clear responses to stimulation of both the ipsilateral and the contralateral retina (Fig. 4 *A*, *B*, *E*). Among individual delayed cells we typically observed slight preferences towards one of the two eyes (Fig. 4 *C*, *E*), with most of these cells exhibiting larger responses to binocular *vs*. monocular (dominant eye) stimulation (Fig. 4 *D*, *E*; mean firing rates during light on). Of note, this binocular integration was not associated with any overt change in sensitivity (F‐tests for difference in EC_50_/Hill slope, *P* = 0.397) and was smaller than a simple linear sum of ipsi‐ and contralateral driven responses (F‐tests, *P* < 0.0001). Response latency (time to peak firing) did not differ significantly between preferred *vs*. non‐preferred eye or binocular stimulation (2.84 ± 0.18 *vs*. 2.94 ± 0.19 and 2.91 ± 0.19, respectively, paired *t*‐tests, both *P* > 0.05).

**Figure 5:**
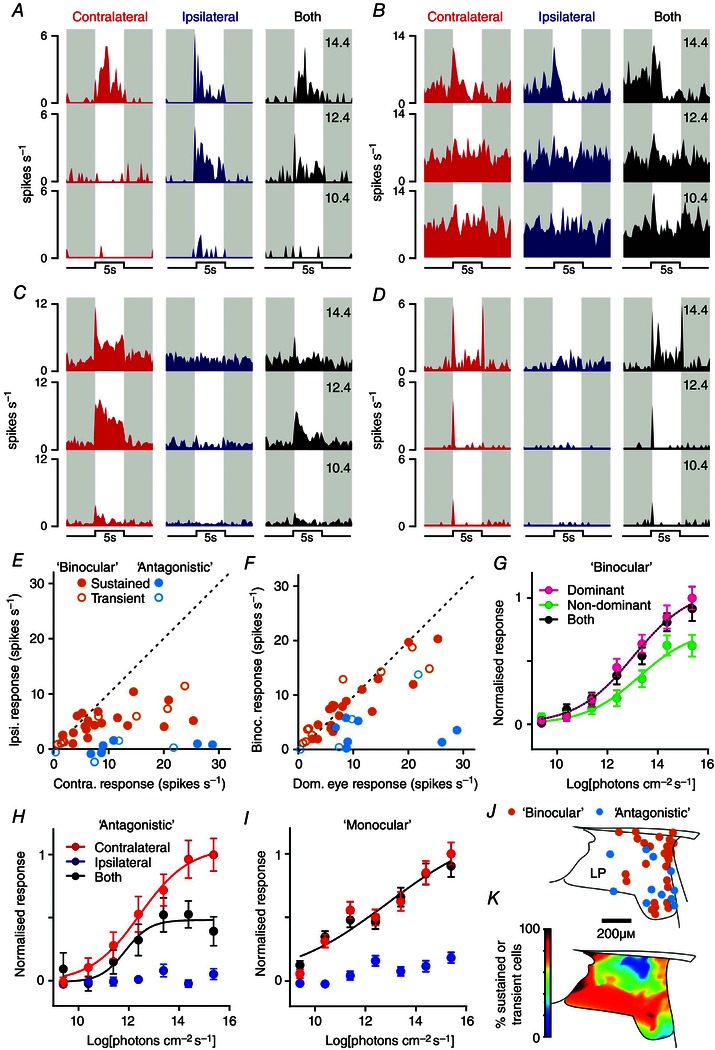
**Subsets of lateral posterior thalamic cells combine eye‐specific signals in a sub‐additive manner** *A–D*, example responses of LP cells following full field light steps applied to the contralateral, ipsilateral or both eyes (5 s, 405 nm LED, 10.4–14.4 log photons cm^–2^ s^–1^). Subsets of sustained and transient LP cells (*n* = 18/67 and 10/89, respectively) exhibited excitatory responses to stimulation of either eye (*A*, *B*), or lacked clear responses to ipsilateral eye stimuli but showed a marked reduction in response under binocular *vs*. contralateral eye only stimulation (*C*, *n* = 7 and 4 for sustained and transient). Remaining sustained and transient cells exhibited purely monocular responses driven by the contralateral eye (*D*) – no cells were observed that exhibited purely ipsilateral eye driven responses. *E*, distribution of contralateral *vs*. ipsilateral eye‐evoked response amplitude (peak spike response observed 0–500 ms after start of light steps between 9.4 and 15.4 log photons cm^–2^ s^–1^) for sustained/transient, binocular or antagonistic cells. *F*, distribution of both *vs*. dominant eye‐only‐evoked responses (measured as in *E*). *G* and *H*, normalised mean (±SEM) initial change in firing (0–500 ms after light step) for ‘binocular’ cells (*G*, *n* = 28), ‘antagonistic’ cells (*H*, *n* = 11) and monocular cells (*I*, *n* = 117) evoked by 9.4–15.4 log photons cm^–2^ s^–1^ light steps applied to the dominant, non‐dominant or both eyes. Note that, for binocular cells (*G*), irradiance response relationships for dominant eye‐only and both eye stimuli were statistically identical whereas for antagonistic cells (*H*) both eye responses were suppressed (four‐parameter sigmoid curve fits with F‐tests, *P* = 0.218 and *P* < 0.0001, respectively). *J*, projected anatomical locations of binocular and antagonistic cells, exhibiting pronounced clustering around the lateral portions of the LP. *K*, proportion of sustained or transient LP cells exhibiting monocular responses as a function of anatomical location, showing highest density in ventral and medial regions.

We next analysed the responses of sustained and transient cells to a similar set of stimuli. The majority of cells in these two groups (67/74 and 89/115 for sustained and transient, respectively) were detected at recording sites located within the LP, with the few remaining cells located laterally and/or ventrally in the APT or Po. Given the relatively low number of cells recorded from these latter regions, we restricted our analysis to cells located in the LP. By contrast with delayed cells, these sustained and transient cells reliably exhibited substantially higher sensitivity (threshold typically 10.4 log photons cm^–2^ s^–1^ under our experimental conditions). Moreover, while all cells could be driven by light steps applied to the contralateral eye, responses to ipsilateral stimuli were much less common than among delayed cells, especially for the transient population (Fig. 5; *n* = 14/89 transient *vs*. *n* = 25/67 for sustained cells; Fisher's exact test, *P* = 0.003).

Where cells did respond to stimulation of the ipsilateral eye, we most commonly observed excitatory responses (Fig. 5 *A*, *B*; *n* = 18 and 10 sustained and transient cells, respectively), with almost all cells exhibiting larger responses to stimulation of the contralateral *vs*. ipsilateral eye (Fig. 5 *E*). In line with the sub‐additive integration previously described in the LGN/V1 (Longordo *et al*. 2013; Zhao *et al*. 2013; Howarth *et al*. 2014), there was no observable change in response magnitude or sensitivity following binocular *vs*. monocular (dominant eye) stimulation under these conditions (Fig. 5 *F*, *G*; F‐test, *P* = 0.22).

A smaller subset of cells exhibited inhibitory binocular interactions (Fig. 5 *C*, *n* = 7 and 4 sustained and transient cells, respectively). These cells all lacked detectable responses to stimulation of the ipsilateral eye in isolation (Fig. 5 *E*, *H*) but exhibited significantly reduced responses to light steps presented to both eyes rather than just the contralateral eye (Fig. 5 *H*). Interestingly, both classes of binocular response were primarily found laterally within LP (Fig. 5 *J*). By contrast, sustained and transient exhibiting pure monocular (contralateral‐driven) responses (Fig. 5 *D*, *I*) were found throughout the LP, but were especially prevalent around ventral and medial portions of the nucleus (Fig. 5 *K*).

### Spatial response properties and retinotopy

**Figure 6:**
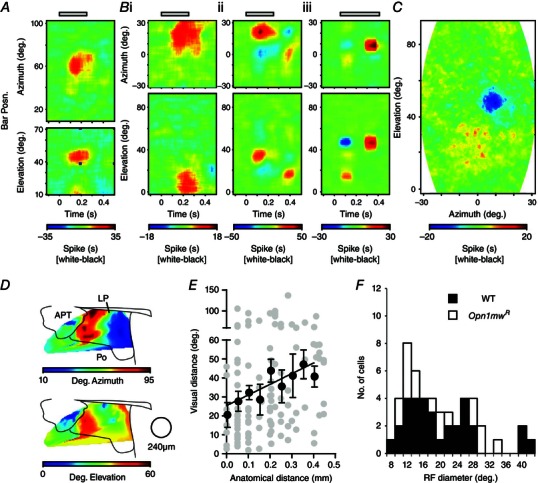
**Retinotopic organisation and RF properties of lateral posterior thalamic neurons** *A* and *B*, spatiotemporal receptive field (RF) maps for four neurons responding to vertical (top panels) or horizontal (bottom panels) flashing bars (250 ms duration), presented to the upper lateral (*A*) or central (*B*) visual field. Colour scale represents the difference in spike rate observed during the presentation of a white *vs*. black bar. Visual angles are expressed relative to the midpoint between the eyes. We observed neurons exhibiting both ON (*A*, *Bi*, *ii*) and OFF RFs (*Biii*), some of which exhibited clear centre surround organisation (*Bii*, *iii*). *C*, two‐dimensional RF map for neuron in *Biii*, in response to sparse noise stimuli (flashing white and black spots), showing good correspondence to results obtained using bars. *D*, mean RF positions in the azimuthal and elevation plane for neurons recorded across the LP and surrounding brain regions, revealing a clear retinotopic order. Data are based on 56 cells (39 WT and 17 *Opn1mw^R^*), binned using a moving circular window (240 μm diameter). *E*, relationship between anatomical separation and RF centre separation for simultaneously recorded cells (linear distance in medial–lateral and dorsal–ventral or azimuth and elevation, respectively). Despite some scatter, there was a clear positive correlation between anatomical separation and difference in RF centre position (*r* = 0.25, *P* = 0.007). *F*, estimated RF size for neurons recorded across the LP region (*n* = 48).

Studies from a range of species indicate that visual inputs to the LP/pulvinar are retinotopically ordered (Petersen *et al*. 1985; Hutchins & Updyke, 1989; Baldwin *et al*. 2011; Li *et al*. 2013). Accordingly, we next set out to determine the extent to which these observations hold true in the mouse, as well as to characterise the RF properties of mouse LP neurons. To this end, in a subset of recordings (*n* = 14 mice), we examined responses to flashing white or black, horizontal or vertical, bars (∼7 deg width), presented across large regions of the visual field (Fig. 6 *A*, *B*; see Methods for further details). In a subset of experiments, we also employed a second sparse noise RF mapping procedure using flashing spots (Piscopo *et al*. 2013; Howarth *et al*. 2014), which produced essentially identical estimates of RF size and position (Fig. 6 *C*; *n* = 9 cells mapped with both).

In total we tested 39 sustained and 78 transient cells with bar stimuli, of which ∼45% (Table 1; *n* = 17 and 36, respectively) exhibited robust spatially localised responses to the appearance of bars in one of the regions we tested. Most of these cells (*n* = 17 and 28) were located within the LP. The remaining neurons (located on the APT border or Po) were excluded from most of the analysis below except for our analysis of retinotopic organisation in Fig. 6 *D*. A further two LP cells from these same experiments that lacked responses to full field light steps (out of 117 cells tested across the LP/Po) also exhibited robust RFs. By contrast, none of the 13 delayed cells tested exhibited any measureable response to the appearance of bars.

**Table 1:** Receptive field properties of cells recorded in the LP and surrounding Po/APT

			Transient					
		Sustained	All	ON	OFF	Delayed	N.R.	All
LP	Mapped/total (%)	17[2]/36 (47.2%)	28/61 (45.9%)	22 [3]/28 (78.6%)	6 [4]/28 (21.4%)	0/9 (0%)	2/90 (2.2%)	53/202 (26.2%)
	Median RF size ± SD	17.4 ± 9.2	18.2 ± 9.2	19.2 ± 8.3	12.7 ± 12.4	N.A.	17.2 & 10.1	17.3 ± 8.9
Po / APT	Mapped /total (%)	0/3 (0%)	8/17 (47.1%)	8/8 (100%)	0/8 (0%)	0/4 (0%)	0/27 (0%)	8/51 (15.7%)
	Median RF size ± SD	N.A.	21.1 ± 8.2	21.1 ± 8.2	N.A.	N.A.	N.A.	21.1 ± 8.2
Total	Mapped/total (%)	17/39 (43.6%)	36/78 (46.2%)	30 [5]/36 (83.3%)	6 [4]/36 (16.7%)	0/13 (0%)	2/117 (1.7%)	55/247 (22.3%)

The table indicates the number of cells where we could map RFs (*vs*. the total number tested) and RF properties, categorised according to responses to full filed light steps. N.R. indicates cells that did not respond to full field stimuli, and numbers in square brackets indicate the subset of cells where we could identify antagonistic surround components.

Among the group of LP cells with mappable RFs, the majority exhibited ON responses (Fig. 6 *A*, *Bi*, *ii*; 41/47 LP cells). All of the cells exhibiting OFF responses (Fig. 6 *Biii*) were classified as transient based on their response to full field light steps. Although antagonistic surrounds are often hard to discern using RF mapping procedures such as ours (Grubb & Thompson, 2003; Piscopo *et al*. 2013; Howarth *et al*. 2014), for some of the sustained and transient cells we recorded (*n* = 9) we could also observe clear evidence of centre surround organisation (Fig. 6 *Bii*, *iii*; Table 1). RFs varied in apparent size but tended to be relatively large (Fig. 6 *F*; Table 1). We did not observe any clear difference in RF sizes between sustained or transient cells (Mann–Whitney test, *P* = 0.83) nor any correlation between RF diameter and eccentricity (*r* = −0.25, *P* = 0.08).

By contrast, we did observe a clear relationship between the projected anatomical locations of recorded cells and RF position at both the single unit (Fig. 6 *D*) and multi‐unit levels (not shown). In particular we found that cells around the lateral borders of the LP encoded nasal regions of visual space, while more medially located cells encoded temporal regions of visual space. Furthermore, this relationship appeared to reverse at the lateral border of the APT. Retinotopic organisation along the dorsal ventral axis was less pronounced, although it appeared from our data that lower elevations may be represented more centrally within the LP and higher elevations more peripherally. As further confirmation that visual inputs to the mouse LP were indeed retinotopically ordered, we exploited the precise geometry of our recording electrodes to examine the relationship between anatomical distance and the separation between RF centres for pairs of simultaneously recorded LP neurons (Fig. 6 *E*). As expected, despite some scatter in the RF centre position among cells sharing relatively close anatomical relationships, this analysis revealed a significant positive correlation between anatomical distance and RF centre separation (*r* = 0.29, *P* = 0.003; *n* = 105 pairs).

## Discussion

To our knowledge, this is the first detailed investigation of visually evoked activity in the mouse LP and Po. Based on responses to simple full field light steps we identify three broad classes of cells which differ with respect to anatomical location and/or photoreceptive origins, suggesting that each group subserves distinct functional roles. As part of this work we therefore provide evidence for a functional melanopsin input to groups of cells in the LP/Po region, extending the known range of central nuclei that receive mRGC signals. Our data also reveal that subgroups of cells with the LP/Po region display various types of binocular interaction and that visual responses within the LP are retinotopically ordered, in line with previous demonstrations in other species and the proposed roles of this nucleus in visual processing (Benevento & Miller, 1981; Petersen *et al*. 1985; Grieve *et al*. 2000).

### Melanopsin influences on the LP/Po

Using anterograde tracers that appeared to preferentially label mRGC target regions, one earlier study suggested mRGCs project to the rat LP and Po (Noseda *et al*. 2010). By contrast, genetically encoded reporters of mRGC projections in the mouse have revealed projections to a region bordering the LP (APT/posterior limitans) but not the LP itself (Hattar *et al*. 2006; Ecker *et al*. 2010; Sakhi *et al*. 2014). Using a highly specific viral tracer, here we confirm that a subset of mRGCs do project to the mouse LP (although we did not find any evidence of direct input to the underlying Po). However, using approaches that enable us to isolate photoreceptor‐specific responses (Brown *et al*. 2010, 2011 *b*; Allen *et al*. 2011, 2014; Walmsley, 2015), we provide evidence that ∼25% of neurons across the LP and dorsal Po are functionally influenced by melanopsin‐derived signals. We should note here that these estimates are based on a subset of recordings performed in *Opn1mw^R^* mice, where > 90% all of the sustained and delayed cells we tested exhibited significantly greater responses to stimuli that preferentially excited melanopsin *vs*. cones. As the proportion of LP/Po cells classified as sustained or delayed in recordings from WT mice was virtually identical to those detected in *Opn1mw^R^* animals, we feel it is safe to assume that the vast majority of such cells also received melanopsin input.

Together then, it appears that melanopsin‐dependent responses are more widespread across the LP/Po than suggested by our anatomical tracing. Insofar as the transduction efficiency of our viral tracer is ∼30%, it is certainly possible that our method is not sensitive enough to reliably detect mRGC inputs to the Po. However, given the convergence of multiple sources of visual input onto these regions (Gale & Murphy, 2014; Morin & Studholme, 2014; Tohmi *et al*. 2014), it is perhaps unsurprising that functional properties might not entirely recapitulate the pattern of direct retinal projections. Indeed, a subset of these melanopsin‐influenced neurons (‘delayed’ cells), mainly found within the Po at the ventral borders of the LP, exhibited very sluggish visual responses akin to those observed in other brain regions that are devoid of direct retinal projections (Brown *et al*. 2011 *b*; Sakhi *et al*. 2014). Thus, delayed cells only respond under high light levels, are preferentially excited by binocular stimuli and lack overt responses driven by rod/cones and to spatially patterned stimuli. Together these properties imply that the (presumably polysynaptic) pathway driving such responses filters out the outer‐retinal signals received by mRGCs (Belenky *et al*. 2003; Wong *et al*. 2007; Schmidt & Kofuji, 2010) and may integrate light intensity across wide regions of space. Indeed, we have previously found evidence that a cortical pathway with analogous properties provides a diffuse binocular brightness signal to the LGN (Howarth *et al*. 2014), suggesting a possible origin for the delayed cell responses we report here. Alternatively, it is formally possible that delayed cells in fact selectively integrate signals originating within defined regions of binocular visual space and that our RF‐mapping stimuli were suboptimal for eliciting these. Future studies using patterned visual stimuli better aligned to the temporal characteristics of melanopsin photoreception (e.g. Procyk *et al*. 2015) should help to distinguish these possibilities.

The second population of melanopsin‐recipient cells (‘sustained’) exhibited properties more consistent with direct mRGC input. These cells displayed rapid rod/cone‐driven responses, as observed in mRGCs and their primary visual targets (Wong *et al*. 2007; Schmidt & Kofuji, 2010; Allen *et al*. 2011, 2014; Brown *et al*. 2011 *b*, 2012) and were enriched in LP regions that exhibited mRGC terminal labelling. Thus, overall, sustained cells comprised ∼40% of the light responsive LP neurons recorded (*vs*. 14% of the population showing delayed responses). If one were to assume that all sustained cells were downstream of mRGCs (which collectively comprise ∼5% of the total RGC population; Ecker *et al*. 2010) this would imply that either a substantial portion of the direct retinal projection to the LP derives from mRGCs and/or that individual mRGCs contact many LP neurons.

An alternative possibility is that either outer retinal and/or melanopsin components of some sustained cell responses also comes via indirect input from the cortex or SC, as suggested for delayed cells. In line with this view, we certainly found a cluster of sustained cells in regions that lack detectable retinal input (Morin & Studholme, 2014). Although the dendritic fields of Po neurons are sufficiently large that they could extend up into the LP (Groh *et al*. 2014), we must conclude that an equally likely explanation then is that at least some sustained cells receive melanopsin input via an indirect route.

### Spatial response properties and occularity

We were able to estimate RF position for a substantial number of sustained and transient neurons across the LP. Consistent with demonstrations in other species (Petersen *et al*. 1985; Hutchins & Updyke, 1989; Baldwin *et al*. 2011; Li *et al*. 2013), we show here that RFs are retinopically ordered across the mouse LP. Thus, nasal regions of visual space are represented laterally and temporal regions more medially within the mouse LP. We did, however, observe some scatter in the relative RF position for cells apparently in close physical proximity. This may reflect the fact that our analysis pools a heterogeneous group of cell types with slightly different retinotopic organisation, but this degree of scatter certainly appears no different from that observed in the mouse LGN (Grubb *et al*. 2003).

In line with the retinotopic arrangement described above, we also found that both sustained and transient cells that responded to full field light steps presented to either eye were strongly clustered around lateral portions of the LP. In cats and monkeys, many LP cells exhibit binocular responses (Benevento & Miller, 1981; Casanova *et al*. 1989; Chalupa & Abramson, 1989). By contrast, binocular responses were relatively rare in the mouse LP (∼25% of sustained and transient cells). This finding is consistent with the sparse ipsilateral retinal projections to the mouse LP (Morin & Studholme, 2014) and presumably reflects the much smaller degree of visual field overlap in this species. Indeed we found broadly similar proportions of cells showing binocular responses in our recent investigation of the mouse dorsal LGN (dLGN) (∼18%; Howarth *et al*. 2014) and in both this earlier study and the present work, in neither region did we find any purely ipsilateral driven cells.

Eye‐specific responses in the mouse LP were typically excitatory, as in cats and primates (Benevento & Miller, 1981; Chalupa & Abramson, 1989), but lacked the additive binocular interactions observed in these other species. Instead, the subadditive responses we observed resembled those seen in the mouse LGN and V1 (Longordo *et al*. 2013; Zhao *et al*. 2013; Howarth *et al*. 2014), possibly reflecting differing roles for binocular integration in rodents (e.g. Wallace *et al*. 2013). We should also note here that, while all binocular cells where we mapped RFs (*n* = 5 cells) specifically responded to stimuli within regions of space visible to both eyes, we did not individually investigate RF locations for ipsilateral and contralateral influences. The extent to which these cells are sensitive to binocular disparity thus remains to be determined. Nonetheless, given that we find retinal projections to the LP are retinotopically ordered, it would be most surprising if these did not originate from at least overlapping regions of visual space, as in the LGN (Howarth *et al*. 2014).

As in primates, most mouse LP neurons (∼80%) exhibited relatively large ON RFs, with inhibitory surrounds only rarely detectible (Mathers & Rapisardi, 1973; Benevento & Miller, 1981). This prevalence of ON responses is somewhat greater than typically reported for the mouse dLGN (∼60% cells), as is the median RF size of LP cells [∼17 *vs*. 11–17 deg for dLGN neurons (Grubb & Thompson, 2003; Piscopo *et al*. 2013)]. These differences could arise either because different populations of RGCs target the LP *vs*. dLGN and/or because these regions receive distinct signals from the cortex and SC (Gale & Murphy, 2014; Tohmi *et al*. 2014). We also found some evidence of heterogeneity with the LP itself, however, as the distribution of RF sizes across our sample appeared multimodal, suggesting the presence of at least two groups of cells with either moderate or very large (>24 deg) RFs. This distinction was not associated with any overt difference in the basic visual response properties, for example sustained or transient [in line with previous reports for dLGN neurons (Grubb & Thompson, 2003)]. Nonetheless, given the typical RF sizes for mouse retinal neurons (<10 deg; Koehler *et al*. 2011), we assume that many of the cells in our sample receive, directly or indirectly, convergent input from multiple RGCs.

Finally, we should note here that within the LP we found a large proportion of cells that failed to respond to any of the visual stimuli we presented (∼50% of cells recorded). By contrast, we have previously found that ∼95% of dLGN cells respond to simple full field light steps (Howarth *et al*. 2014). It is possible then that many cells in the mouse LP are concerned with functions other than visual processing. In line with this view, we know that in primates, only a portion of the pulvinar receives visual signals, with medial and anterior regions implicated in somatosensory/multisensory processing (Kaas & Lyon, 2007). In the mouse, we found that light‐insensitive neurons were most prevalent in central/ventral regions of the LP but not overtly segregated from visually responsive cells.

An alternative possibility, then, is that a substantial proportion of the LP cells we recorded are insensitive to simple visual stimuli and instead respond only to more complex visual features. Indeed, reports from other species indicate that many LP cells are sensitive to simple or complex motion or respond to specific shapes (Godfraind *et al*. 1969; Benevento & Miller, 1981; Petersen *et al*. 1985; Chalupa & Abramson, 1989; Casanova & Molotchnikoff, 1990; Merabet *et al*. 1998; Boire *et al*. 2004; Billington *et al*. 2011; Van Le *et al*. 2013). In line with the idea that some mouse LP cells might also preferentially respond to moving stimuli, a recent investigation indicates that a major source of SC input to the LP comes via cells which preferentially respond to very small slowly moving objects (Gale & Murphy, 2014). Similarly, SC inputs to the mouse LP have been implicated in triggering defensive responses that are very selective for specific stimulus features (an overhead expanding shadow; Yilmaz & Meister, 2013; Wei *et al*. 2015). In this regard then, we speculate that the visual stimuli we have employed in this study may preferentially identify a subset of LP cells receiving direct rather than purely indirect visual inputs. This possibility is consistent with the overall similar response latency we observe for sustained and transient cells between the LP and LGN (Howarth *et al*. 2014), in contrast to findings in the cat (using more complex stimuli) that LP responses tend to be slower than those of LGN cells (Cai *et al*. 1997; Ouellette & Casanova, 2006; Moore *et al*. 2014; Piché *et al*. 2015).

### Functional subdivisions

In primates and cats the visual pulvinar/LP is functionally subdivided on the basis of cortical and subcortical inputs into a medial portion, concerned with control of visually guided actions, and a lateral portion involved in object vision (Caldwell & Mize, 1981; Boire *et al*. 2004; Kaas & Lyon, 2007). Although the mouse LP has also been subdivided into medial and lateral portions (Paxinos, 2001), the precise cytoarchitectural boundaries between these regions are hard to discern in this species, nor do patterns of retinal, SC or cortical connectivity suggest any overt functional distinction between medial and lateral aspects (Gale & Murphy, 2014; Morin & Studholme, 2014; Tohmi *et al*. 2014). In line with this arrangement, we certainly found no clear evidence of medial–lateral differences in the basic visual response properties of LP cells. Indeed, our RF mapping suggests the presence of a continuous retinotopic azimuth map running across the full extent of the LP (at least at the mid‐caudal level). Thus, while we find that cells with binocular responses are strongly clustered along the lateral margins of the LP, this finding is simply explained by the fact that more medial portions process signals originating from regions of visual space that are not visible to both eyes.

Nonetheless, while the basic visual response properties we describe here do not neatly segregate according to traditional anatomical divisions, we certainly find a range of visual response properties across the LP and underlying Po that suggest some functional specialisations. Thus, traditionally cells with ‘transient’ responses have been implicated in high temporal acuity vision, suggesting the transient cells identified here in the mouse LP could play a role in processing motion‐related information (Tohmi *et al*. 2014). By contrast, cells with ‘sustained’ responses may be better suited for a role in object vision. In this regard it is noteworthy that we find sustained cells at highest density across more dorsal portions of the LP which seem to preferentially connect to V1 as opposed to higher visual areas (Tohmi *et al*. 2014). Moreover, our retinotopic mapping data also hint at the possibility that the elevation axis could be separately represented for dorsal and ventral potions of the LP.

Projections from both the LP and the Po to the amygdala have been implicated in regulating various kinds of reflex responses to threatening stimuli, at least one of which (light aversion in neonatal mice) is heavily dependent on melanopsin photoreception (Morris *et al*. 1999; Goossens *et al*. 2007; Delwig *et al*. 2012; Van Le *et al*. 2013; Wei *et al*. 2015). Accordingly, sustained and delayed cells within Po or LP appear well placed to contribute to such subcortical visual responses. It is also noteworthy that light‐induced suppression of locomotor activity in adult mice (negative masking) is also strongly dependent on melanopsin photoreception (Mrosovsky & Hattar, 2003). Although the central circuits mediating such responses are unknown, our identification of mRGC inputs to the LP provides a new possible origin for such behaviour.

Finally, the LP and Po are known sites of multisensory integration (Avanzini *et al*. 1980; Mooney *et al*. 1984; Noseda *et al*. 2010; Groh *et al*. 2014). Thus, a subset of LP/Po cells are both responsive to nociceptive (stimulation of the dura) and visual stimuli (Noseda *et al*. 2010) and are believed to contribute to light‐induced exacerbation of migraine pain. Given the very sluggish visual responses of the multisensory neurons identified in this earlier study, we suggest that such cells correspond to the population of delayed cells identified here.

In conclusion, we demonstrate the presence of a variety of visual response types within the mouse LP and Po whose basic functional properties and organisation are compatible with the putative roles of the nuclei in visual processing and provide a potential origin for various reflex responses to light.

## Additional information

### Competing interests

None declared.

### Author contributions

T.M.B., A.E.A. and C.A.P. designed the experiments, A.E.A., C.A.P., M.H. and L.W. collected and analysed the data, and T.M.B. and A.E.A wrote the manuscript. All authors approved the final version of the manuscript, agree to be accountable for all aspects of the work and all persons designated as authors qualify for authorship. All experiments were performed at the University of Manchester, UK.

### Funding

This work was funded by a Biotechnology and Biological Sciences Research Council (BBSRC, UK) David Phillips fellowship to T.M.B. (BB/I017836/1); A.E.A. and C.A.P. were funded by the BBSRC (BB/I007296/1) and European Research Council (ERC; 268970 to Robert Lucas, University of Manchester).
